# Loss of SELENOW aggravates muscle loss with regulation of protein synthesis and the ubiquitin-proteasome system

**DOI:** 10.1126/sciadv.adj4122

**Published:** 2024-09-20

**Authors:** Jia-Cheng Yang, Meng Liu, Rong-Hui Huang, Ling Zhao, Qin-Jian Niu, Ze-Jing Xu, Jin-Tao Wei, Xin Gen Lei, Lv-Hui Sun

**Affiliations:** ^1^ State Key Laboratory of Agricultural Microbiology, Hubei Hongshan Laboratory, Frontiers Science Center for Animal Breeding and Sustainable Production, College of Animal Science and Technology, Huazhong Agricultural University, Wuhan, Hubei 430070, China.; ^2^ Institute of Animal Husbandry and Veterinary Sciences, Hubei Academy of Agricultural Sciences, Wuhan 430064, China.; ^3^ Department of Animal Science, Cornell University, Ithaca, NY 14853, USA.

## Abstract

Sarcopenia is characterized by accelerated muscle mass and function loss, which burdens and challenges public health worldwide. Several studies indicated that selenium deficiency is associated with sarcopenia; however, the specific mechanism remains unclear. Here, we demonstrated that selenoprotein W (SELENOW) containing selenium in the form of selenocysteine functioned in sarcopenia. SELENOW expression is up-regulated in dexamethasone (DEX)–induced muscle atrophy and age-related sarcopenia mouse models. Knockout (KO) of SELENOW profoundly aggravated the process of muscle mass loss in the two mouse models. Mechanistically, *SELENOW* KO suppressed the RAC1-mTOR cascade by the interaction between SELENOW and RAC1 and induced the imbalance of protein synthesis and degradation. Consistently, overexpression of SELENOW in vivo and in vitro alleviated the muscle and myotube atrophy induced by DEX. SELENOW played a role in age-related sarcopenia and regulated the genes associated with aging. Together, our study uncovered the function of SELENOW in age-related sarcopenia and provides promising evidence for the prevention and treatment of sarcopenia.

## Teaser

SELENOW deficiency aggravates sarcopenia by impairing proteostasis in skeletal muscle.

## INTRODUCTION

Sarcopenia is characterized by a substantial reduction of skeletal muscle mass and strength, and it commonly occurs as an age-related process and affects almost all elderly people (*1*). Sarcopenia can cause poor functional status associated with increased adverse outcomes including falls, frailty, and mortality, and its risk increases with age (*2*). Although exercise is undoubtedly the most effective approach to reverse sarcopenia (*3*), it is not always applicable, particularly for older adults. Therefore, the development of strategies to address or mitigate the loss of skeletal muscle mass is urgently needed. In this regard, nutritional strategies have drawn much attention (*4*, *5*).

Selenium is an essential nutrient for humans and animals and plays pivotal roles in antioxidant defense, anticancer, and anti-inflammatory effects (*6*–*8*). Many studies have shown that selenium deficiency induces nutritional skeletal muscle disorders (*9*), such as muscular dystrophy in several animals (*10*–*12*) and diseases like SELENON-related myopathy (*13*) and Keshan disease in humans (*14*). In addition, selenium concentration was reported to be positively associated with muscle mass and strength in older adults (*15*, *16*), which implies that selenium is related to the development of age-related sarcopenia. In addition, numerous studies have demonstrated that selenium supplementation is an important strategy to prevent aging-related diseases (*17*); the underlying mechanism is not clear. Despite the biological functions of selenium, attributed primarily to its presence as selenocysteine (Sec) in 25 selenoproteins (*18*, *19*), which selenoproteins are involved in age-related sarcopenia are still unclear.

According to the gene expression signature in rats (*20*), among all selenoproteins, selenoprotein W (SELENOW) was the only one identified in the age-related genes. SELENOW is the smallest 9.5-kDa selenoprotein, has a thioredoxin-like fold, and belongs to the Rdx family, which implicates exerting important physiological functions during the aging process (*21*–*23*). It shows the highest expression in the skeletal muscle (*24*, *25*) and was the first selenoprotein described to be linked to white muscle disease in lambs (*26*). Over the past decades, numerous in vitro studies have demonstrated that SELENOW can function in redox regulation (*27*–*30*), cell cycle progression (*31*–*33*), and myogenic differentiation (*34*–*36*), which are closely related to muscle growth and development. However, the in vivo function of SELENOW in skeletal muscle, especially in age-related sarcopenia, remains unknown.

Here, we generated *SELENOW* knockout (KO) mice to determine the *vivo* function of SELENOW in skeletal muscle. We found that (i) *SELENOW* KO aggravated muscle mass loss in dexamethasone (DEX)–induced muscle atrophy and age-related sarcopenia mouse models; (ii) *SELENOW* KO disrupted the balance of protein synthesis and degradation in skeletal muscle through the RAC1-mTOR cascade; and (iii) overexpression of SELENOW alleviated DEX-induced muscle and myotube atrophy in in vivo and in vitro studies. These results provide a potential intervention target to prevent and treat sarcopenia.

## RESULT

### SELENOW is up-regulated in skeletal muscle of mice with atrophy and sarcopenia

**Fig. 1.**
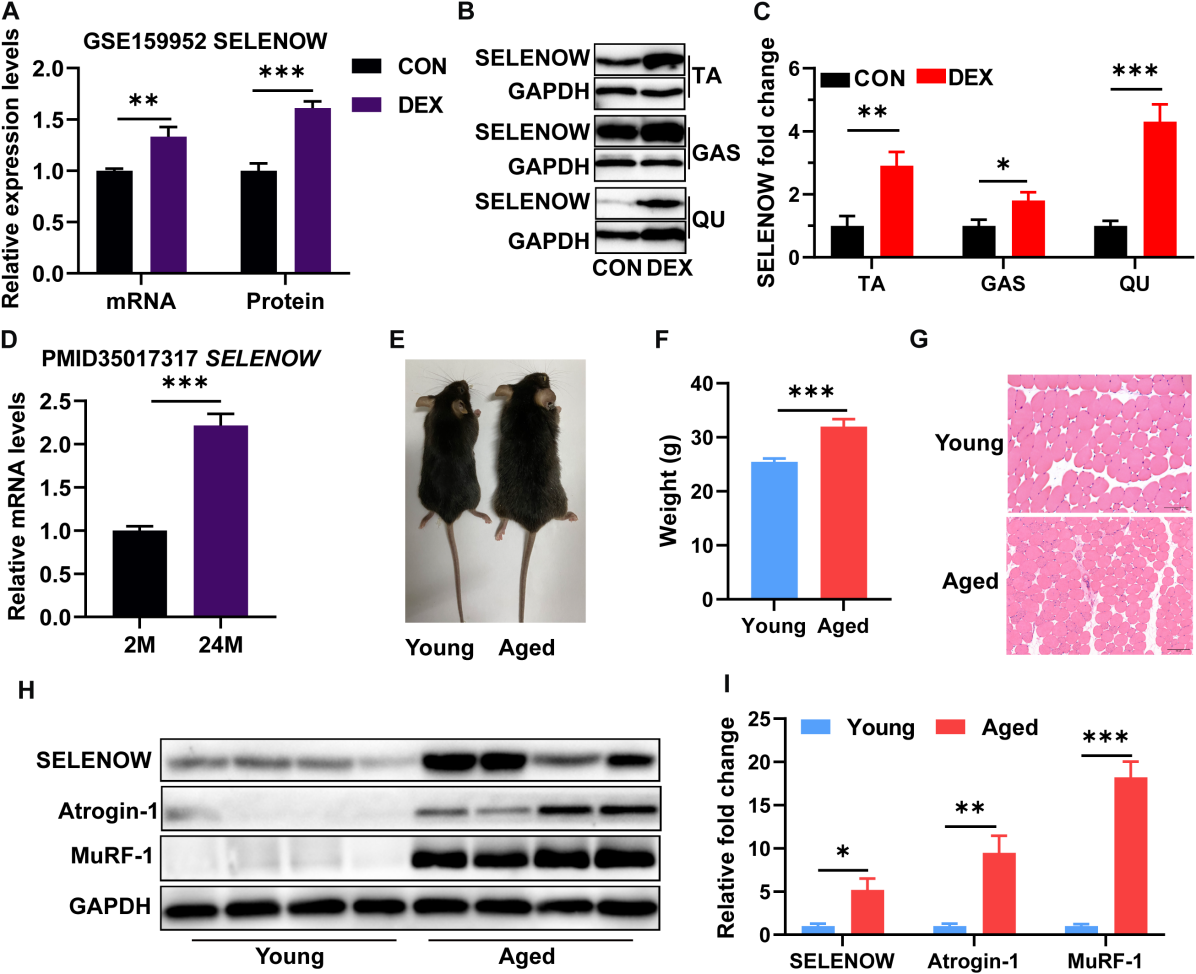
SELENOW is up-regulated in muscle with age-related sarcopenia. (**A**) Relative expression levels of SELENOW in mRNA and protein level of GAS muscle from the GSE159952 database, *n* = 4. (**B** and **C**) Western blot analysis of SELENOW expression in TA, GAS, and QU muscles after DEX treatment, *n* = 4. (**D**) Relative expression levels of Fndc5 in mRNA and protein level of GAS muscle from the PMID 35017317 paper, *n* = 5 to 6. (**E**) Photographs of young (3-month-old) and aged (24-month-old) mice. (**F**) Body weights of young and aged mice, *n* = 12 to 13. (**G**) Representative hematoxylin and eosin (H&E)–stained cross sections in TA muscle of young and aged mice; scale bars, 100 μm. (**H** and **I**) Western blot analysis of protein levels of SELENOW, Atrogin-1, and MuRF-1 in TA muscle of young and aged mice. GAPDH was used as the loading control, *n* = 4. Data are means ± SEM. Student’s *t* test, *P ≤ 0.05, **P ≤ 0.01, ***P ≤ 0.001

Because of the possible roles of SELENOW in sarcopenia, we identified the expression of SELENOW in skeletal muscle of atrophy and sarcopenia mice. We first analyzed the Gene Expression Omnibus (GEO) database (GSE 159952) and found that SELENOW mRNA and protein expression were up-regulated in gastrocnemius (GAS) muscles of DEX-induced muscle atrophy mice (Fig. 1A). Here, as compared with the control group, we also found that SELENOW was up-regulated in tibialis anterior (TA), GAS, and quadricep (QU) muscle protein level after DEX treatment (Fig. 1, B and C). Subsequently, we analyzed the data of PMID 35017317 and found that *SELENOW* mRNA expression was up-regulated in hindlimb skeletal muscle of aging-induced sarcopenia mice (Fig. 1D). To establish chronic sarcopenia mice, we raised wild-type (WT) C57 mice until they were 24 months old. As compared with young mice (3 months old), aged mice (24 months old) showed thinner and bleaker hair (Fig. 1E), much heavier body weight (Fig. 1F), reduced fiber size of TA muscle (Fig. 1G), and increased atrophic gene (Atrogin1 and MuRF1) expression in TA muscle (Fig. 1, H and I), indicating the presence of sarcopenia in aged mice. Of note, SELENOW was also up-regulated in the TA muscle protein level of aged mice (Fig. 1, H and I). Overall, these data suggest an increased level of SELENOW in sarcopenia.

### SELENOW is dispensable for bodyweight and muscle mass maintenance in basal conditions

**Fig. 2.**
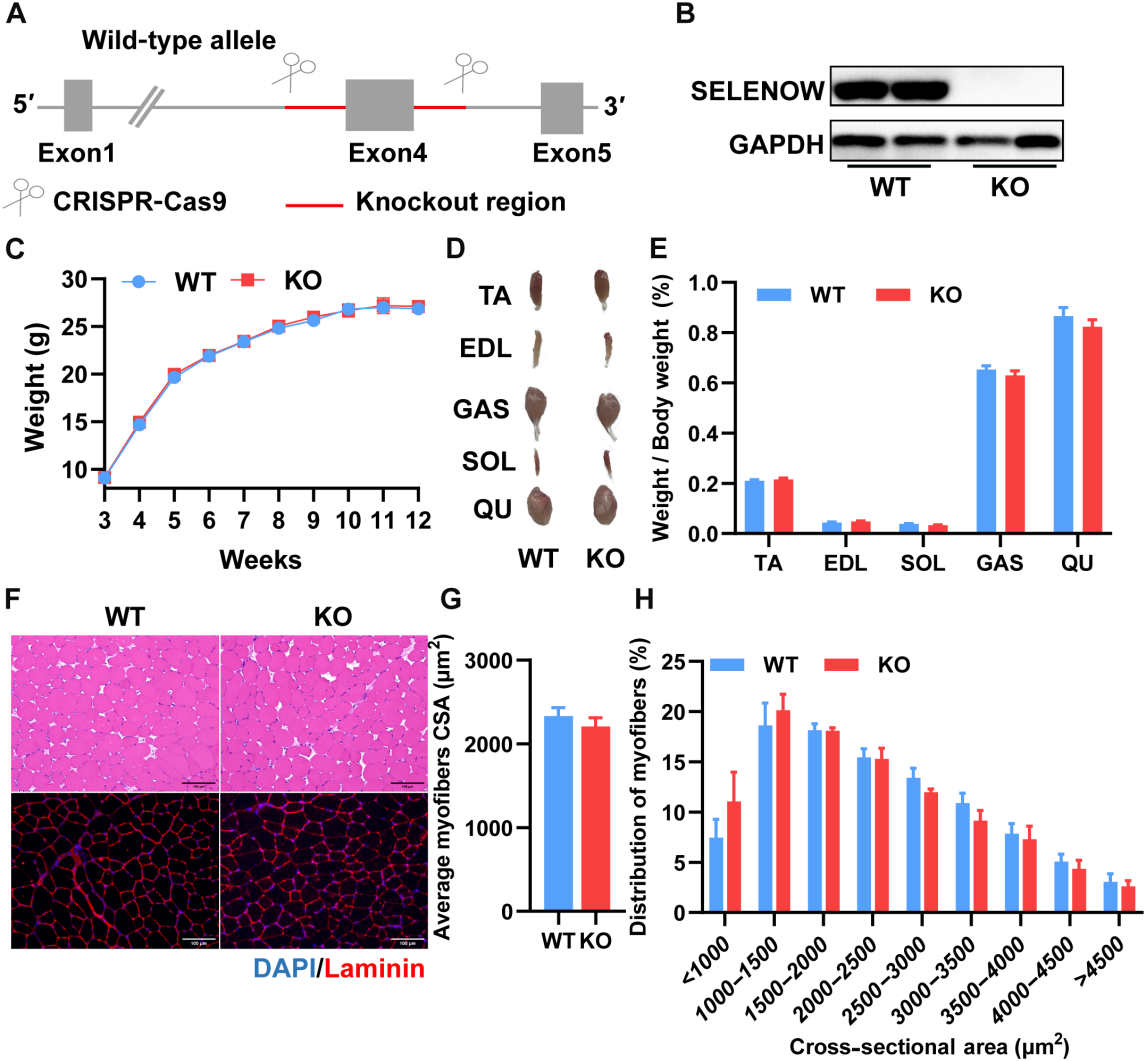
SELENOW deletion does not affect muscle size or histology. (**A**) CRISPR-Cas9 gene-targeting strategy for the generation of SELENOW KO mice. (**B**) Western blot analysis of SELENOW in TA muscles of WT and KO mice, *n* = 2. (**C**) Average body weights of mice from 3 to 12 weeks of age, *n* = 7. (**D** and **E**) Gross morphology mass analysis of TA, EDL, GAS, SOL, and QU in WT and KO mice, *n* = 7. (**F**) Representative H&E staining and Laminin staining of the myofiber cross section of TA; scale bars, 100 μm. (**G** and **H**) Related myofiber cross-sectional area (CSA) and frequency of distribution for CSA of TA muscle, *n* = 7. Data are means ± SEM; Student’s *t* test.

To identify the specific function of SELENOW in skeletal muscle, we generated *SELENOW* KO mice by deleting exon 4 with two sgRNAs (Fig. 2A), and Western blot confirmed that SELENOW has been ablated in skeletal muscle (Fig. 2B). As compared with WT mice, there was no bodyweight or muscle mass change from weaning to 12 weeks (Fig. 2, C to E). To assess the role of SELENOW in muscle development, maintenance, and physiology, histological and immunofluorescence analyses were used in TA muscle. Hematoxylin and eosin (H&E) staining of muscle-detected normal muscle architecture with no inflammation, compensatory muscle regeneration, and degeneration in KO muscles (Fig. 2F), and laminin B1 staining showed the cross-sectional area (CSA) of myofiber distribution no different between WT and KO muscle (Fig. 2, G and H). These results indicated that SELENOW is dispensable for skeletal muscle growth and maintenance in the basal condition.

### *SELENOW* KO aggravates DEX-induced muscle atrophy in mice

**Fig. 3.**
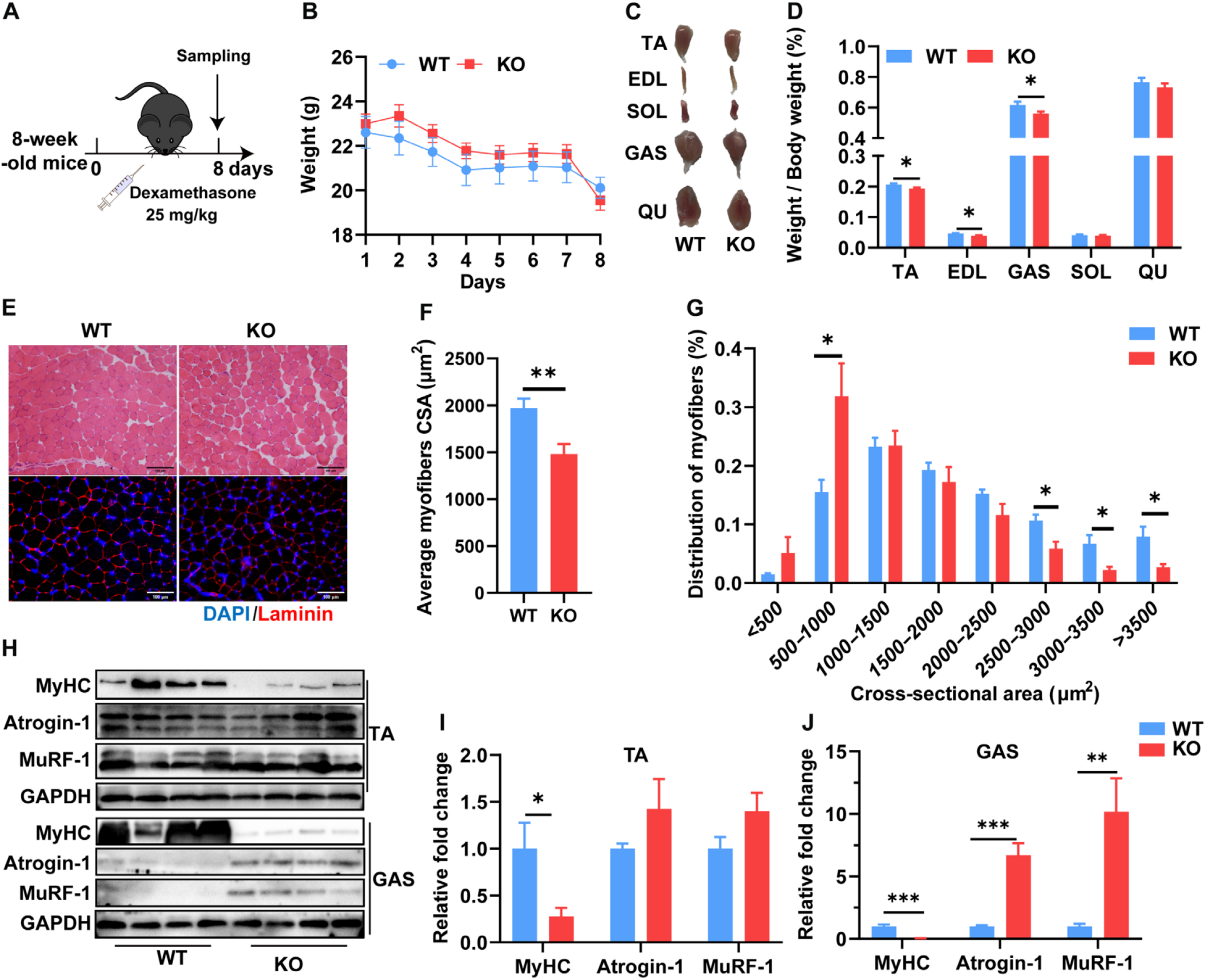
SELENOW deletion aggravates DEX-induced muscle atrophy. (**A**) Schematic showing the acute sarcopenia mice model established by the treatment of DEX in 8-week-old mice for 8 days. (**B**) Comparison of DEX treatment on body weight in WT and KO mice, *n* = 8. (**C** and **D**) Gross morphology mass analysis of TA, EDL, Gas, SOL, and QU in mice after DEX treatment, *n* = 8. (**E**) Representative H&E staining and Laminin staining of myofiber cross section of TA after DEX treatment; scale bars, 100 μm. (**F** and **G**) Related myofiber CSA and frequency of distribution for CSA of TA muscle after DEX treatment, *n* = 8. (**H** to **J**) Western blot analysis of protein levels of MyHC, Atrogin-1, and MuRF-1 in TA and Gas muscle. GAPDH was used as the loading control, *n* = 4. Data are means ± SEM; Student’s *t* test, *P ≤ 0.05, **P ≤ 0.01, ***P ≤ 0.001.

Given the increased level of SELENOW in atrophy muscle, we next investigated the function of SELENOW in the DEX-induced muscle atrophy mouse model. As a previous study described (*37*), 2-month-old male mice were treated with DEX for 8 days via intraperitoneal injections of 25 mg kg^−1^ day^−1^ (Fig. 3A). Compared to WT mice, KO mice body weight did not show a notable change during DEX treatment (Fig. 3B), whereas the TA, extensor digitorum longus (EDL), and GAS muscle index were lower in KO mice (Fig. 3, C and D). The H&E and laminin B1 staining of TA muscle were performed to characterize the effect of SELENOW in muscle atrophy (Fig. 3E). After statistical analysis of fiber size, we found that KO muscle fibers displayed substantially smaller diameters (Fig. 3, F and G). In addition, MyHC was notably decreased in TA and GAS muscle, whereas atrophic genes (Atrogin-1 and MuRF-1) were strongly up-regulated in GAS muscle (Fig. 3, H to J). Collectively, these results suggest that *SELENOW* KO aggravates muscle atrophy and increases the rate of protein ubiquitination in DEX-induced muscle atrophy of mice.

### *SELENOW* KO worsens the degree of aging-related sarcopenia in mice

**Fig. 4.**
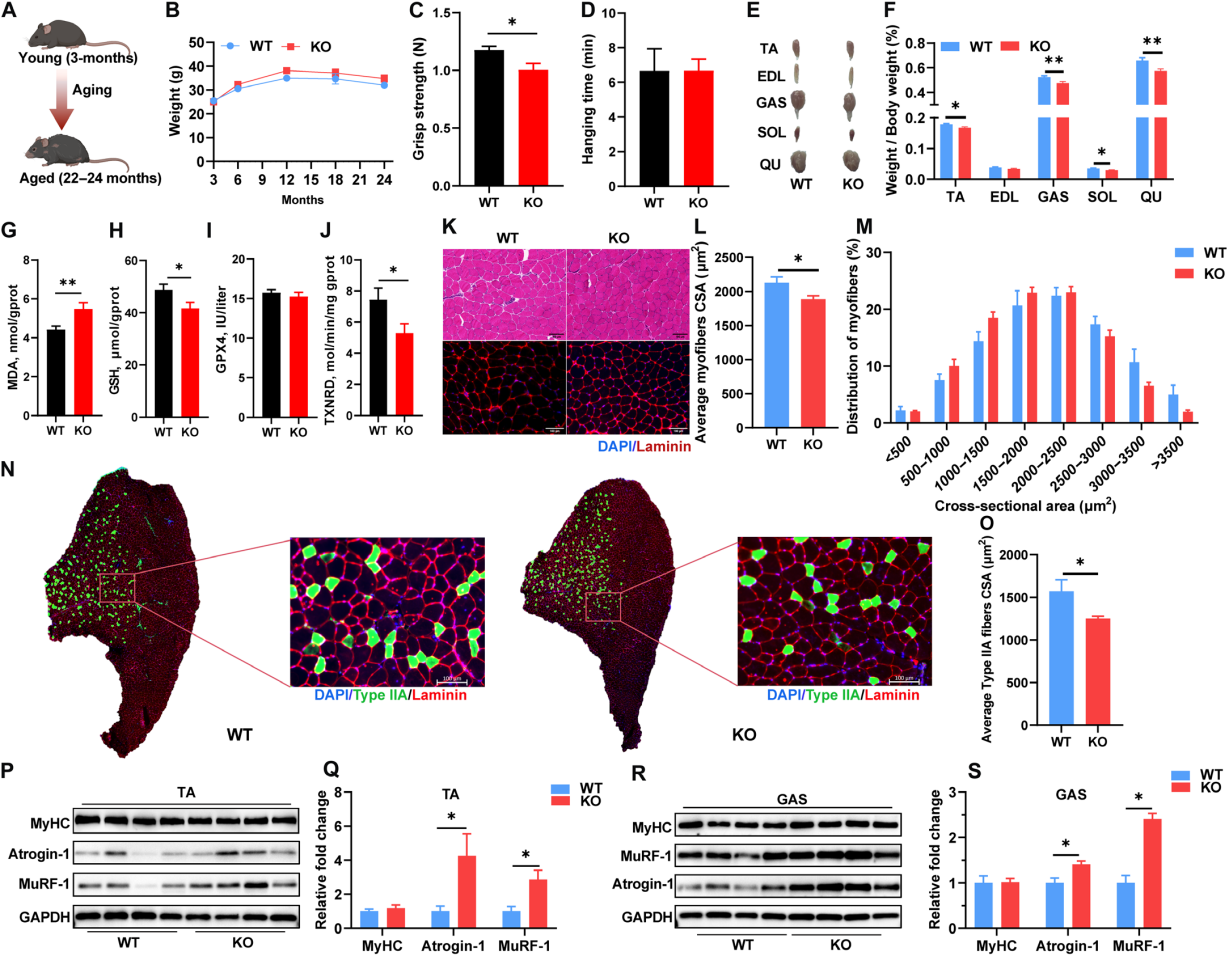
SELENOW deletion aggravates age-related sarcopenia. (**A**) Schematic showing the chronic sarcopenia mice model established by raising C57 mice from 3 months to 22 to 24 months. (**B**) Average body weights of mice from 3 to 24 months of age, *n* = 13 to 15. (**C** and **D**) Grip strength and hanging time of C57 mice from 22 to 24 months of age, *n* = 8 to 9. (**E** and **F**) Gross morphology mass analysis of TA, EDL, GAS, SOL, and QU in aged mice after DEX treatment, *n* = 18 to 22. (**G** to **J**) MDA, GSH, GPX4, and TXNRD concentration or activity in GAS muscle, *n* = 13 to 15. (**K**) Representative H&E staining and Laminin staining of myofiber cross section of TA muscle; scale bars, 100 μm. (**L** and **M**) Related myofiber CSA and frequency of distribution for CSA of TA muscle a, *n* = 7. (**N** and **O**) Representative Laminin staining of type IIA fibers in TA muscle; scale bar, 100 μm. (O) Related CSA of type IIA fibers in TA muscle, *n* = 5. (**P** to **S**) Western blot analysis of protein levels of MyHC, Atrogin-1, and MuRF-1 in TA and GAS muscle. GAPDH was used as the loading control, *n* = 4. Data are means ± SEM; Student’s *t* test, *P ≤ 0.05, **P ≤ 0.01.

To further determine the effects of SELENOW on muscle mass and function in sarcopenia, 22- to 24-month-old mice were used as a naturally chronically occurring aging-associated sarcopenia model (Fig. 4A). Similar to the DEX-induced muscle atrophy of mice model, there was no notable change bodyweight change between WT and KO mice from 3 to 24 months (Fig. 4B). As compared with WT mice, KO mice also showed weaker grip strength, whereas there was no change in hanging time (Fig. 4, C and D). In addition, the weight of TA, GAS, soleus (SOL), and QU muscles were notably lower in KO mice (Fig. 4, E and F). Correspondingly, *SELENOW* KO slightly reduced glucose clearance ability in aged mice, as illustrated by a higher blood glucose concentration at the 15-min point after insulin injection (fig. S1). Given SELENOW’s established role in redox control, we investigated whether its ablation affects the status of the redox environment. *SELENOW* KO led to an increased malondialdehyde (MDA) concentration and a decrease in glutathione (GSH) level and thioredoxin reductase (TXNRD) activity in GAS muscle (Fig. 4, G to J), indicating that SELENOW deletion reduced the antioxidant capacity.

As compared with WT mice, KO mice had smaller mean CSA of individual myofibers in TA muscle of aged mice (Fig. 4, K to M), and similarly in EDL, SOL, GAS, and QU muscles (fig. S2). Given the up-regulation of SELENOW in slow fibers during aging (*38*), we also texted the slow fiber in TA muscles. *SELENOW* KO mice exhibit a reduced size of type IIA fibers in the TA muscle (Fig. 4, N and O), while type I fibers are rarely found in TA muscle (fig. S3). Of note, Atrogin-1 and MuRF-1 were up-regulated in TA and GAS muscles, whereas MyHC did not change in TA or GAS muscles (Fig. 4, P to S). Altogether, these results further demonstrated that *SELENOW* KO also aggravated muscle loss in aging-induced sarcopenia mice and induced the imbalance of protein homeostasis.

### *SELENOW* KO induces an imbalance of proteostasis through rho signaling

**Fig. 5.**
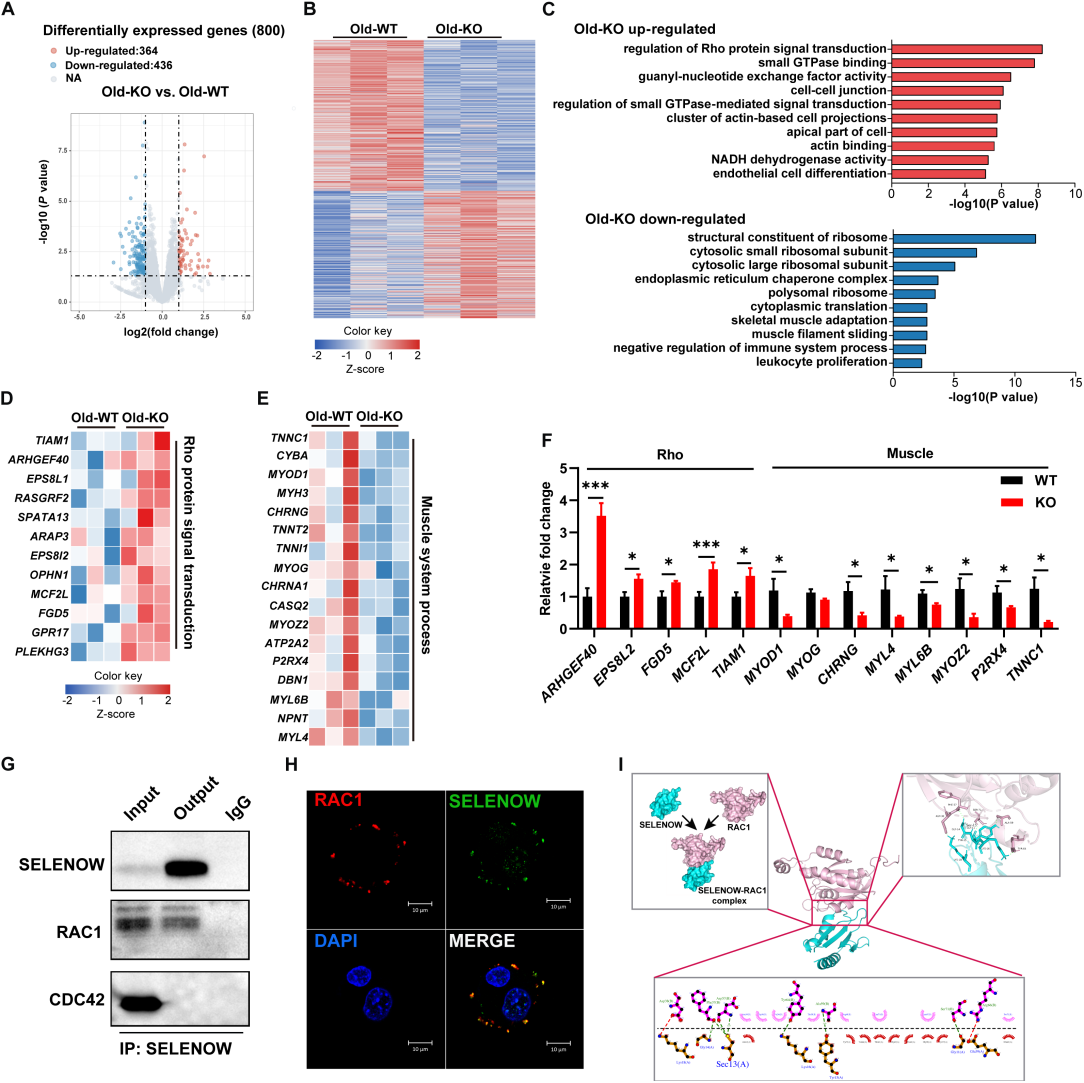
SELENOW functions in sarcopenia may via Rho signaling. (**A**) Volcano diagram of DEGs in TA muscle between WT and KO aged mice, *n* = 3. (**B**) Heatmap diagram showing the cluster analysis of DEGs in TA muscle of WT and KO aged mice, *n* = 3. (**C**) GO analyses showing the enrichment of functional categories in TA muscle of WT and KO aged mice, *n* = 3 mice. (**D** and **E**) Heatmap diagram showing the up-regulated genes related to small Rho protein signaling translation and down-regulated genes related to muscle system processes in TA muscle of WT and KO mice, *n* = 3. (**F**) qPCR analysis of the relative mRNA expression of Rho transduction related gene (*ARHGEF40*, *EPS8L2*, *FGD5*, *MCF2L*, and *TIAM1*) and muscle system-related genes (*MYOD*, *MYOG*, *CHRNG*, *MYL4*, *MYOZ2*, *P2RX4*, and *TNNC1*), *n* = 6. (**G**) Co-IP analysis the protein interaction between Rho protein (RAC1 and CDC42) and SELENWO. (**H**) Laser confocal showing the cellular localization of RAC1 and SELENOW in C2C12 cells. (**I**) The binding mode prediction between SELENOW and RAC1. Data are means ± SEM; Student’s *t* test, *P ≤ 0.05, ***P ≤ 0.001.

To explore the mechanism underlying the KO of SELENOW aggravate sarcopenia in mice, RNA sequencing analysis was performed with TA muscle in aged mice. Overall, 14,124 genes were detected. Differentially expressed genes (DEGs) were identified as *P* < 0.05 and fold change >1.5 or <0.7. Here, 436 genes were notably up-regulated, whereas 364 genes were notably down-regulated in KO aged mice muscle (Fig. 5A). Heatmap cluster analysis nicely distinguished the expression of different expressions of DEGs between WT and KO mice (Fig. 5B). We found that 6.6% of the DEGs in the skeletal muscle of aging mice between WT and KO were aging-related genes (table S1 and fig. S4A). To further unravel the function of DEGs, Gene Ontology (GO) function analysis and Kyoto Encyclopedia of Genes and Genomes (KEGG) pathways analysis were used. According to the GO analysis, the top 10 enriched GO pathways of the up-regulated DEGs were mainly enriched in small GTPase (guanosine triphosphatase) binding, Rho and Ras protein signal transduction, NADH dehydrogenase activity, and the immune effector process, whereas the top 10 GO enriched pathways of down-regulated DEGs were mostly associated with muscle system processes, negative regulation of immune system process, and tumor necrosis factor production (Fig. 5C). Similarly, the top 25 enriched KEGG pathways showed the DEGs up-regulated in Rho GTPase signaling (cGMP-PKG signaling pathway and cAMP signaling pathway), but down-regulated in proteostasis (ribosome and lysosome) (fig. S4, B and C). In addition, although not immediately apparent, KEGG enrichment analysis of DEGs also revealed that loss of SELENOW influences the regulation of the reactive oxygen species (ROS) metabolic process. On the basis of the GO analysis, we also generated a heatmap to show the expression of genes enriched in the muscle system process and Rho protein signal transduction (Fig. 5, D and E). Notably, the real-time q-PCR result showed that mRNA levels of genes (*ARHGEF40, EPS8l2, FGD5, MCF2L,* and *TIAM1*) associated with Rho signaling transduction were increased and genes (*MYOD1, MYOG, CHRNG*, *MYL4*, *MYL6B*, *MYOZ2, P2RX4,* and *TNNC1*) associated with muscle system processes were decreased in KO muscle, which is in agreement with the RNA sequencing (RNA-seq) results (Fig. 5F).

Subsequently, we analyzed the data of the proteins interacting with SELENOW (*39*). The GO enrichment analysis showed that these proteins are associated with protein translation synthesis, protein ubiquitin degradation, and GTPase transduction (fig. S5). Here, we also investigated the protein interaction between SELENOW and Rho GTPase (RAC1 and CDC42). The co-immunoprecipitation (Co-IP) assay demonstrated that SELENOW interacts with RAC1 (Fig. 5G), and laser confocal microscopy showed the co-location of SELENOW and RAC1 in muscle cells (Fig. 5H). In addition, the binding mode prediction illustrated that SELENOW binds with RAC1 by two ionic bonds and seven hydrogen bonds, and three binding sites occurred in Sec^13^ of SELENOW (Fig. 5I). These results indicated that SELENOW’s function in proteostasis may be via Rho protein signal transduction, especially RAC1 signaling.

### *SELENOW* KO induces an imbalance of proteostasis possibly via the RAC1-mTOR cascade

**Fig. 6.**
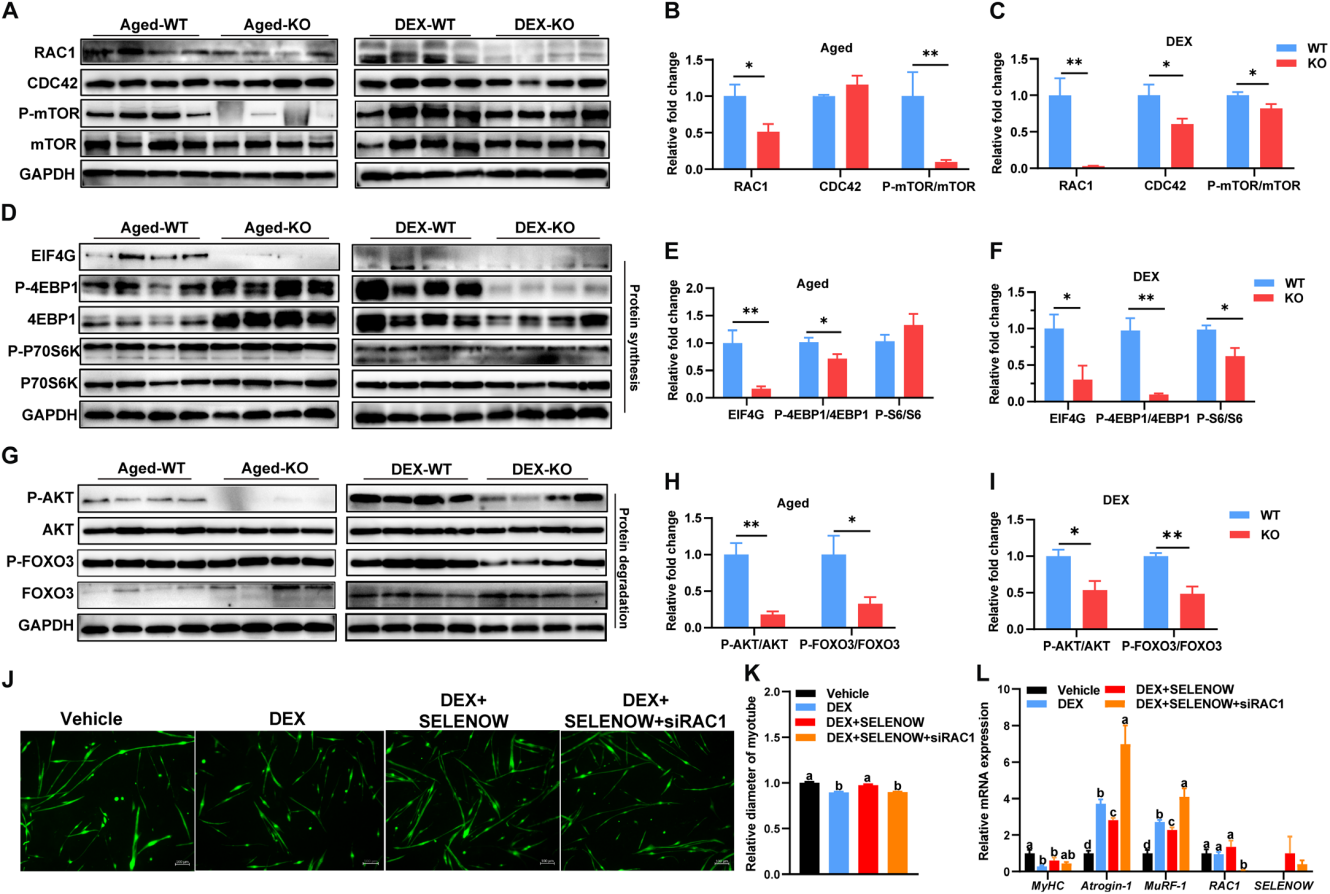
SELENOW deletion effects on proteostasis via the RAC1-mTOR cascade. (**A** to **C**) Western blot analysis of protein levels of RAC1, AT1, CDC42, mTOR, and P-mTOR (S2248) in GAS muscle of the sarcopenia mice model, GAPDH was used as the loading control, *n* = 4. (**D** to **F**) Western blot analysis for the protein synthesis pathways [EIF4G, 4EBP1, P-4EBP1(T45), P70S6K, and P-P70S6K (S434)] in GAS muscle of the sarcopenia mice model, GAPDH was used as loading control, *n* = 4. (**G** to **I**) Western blot analysis for the protein degradation pathways [AKT, P-AKT (S473), FOXO3, and P-FOXO3 (S253)] in GAS muscle of the sarcopenia mice model; GAPDH was used as the loading control, *n* = 4. (**J**) Immunofluorescent staining of MyHC in primary myotubes. Before DEX treatment, the primary myotubes were infected with a control vehicle, a SELENOW vehicle, or a combination of SELENOW vehicle and siRAC1 mimic; scale bar, 100 μm. (**K**) Quantification of these primary myotube diameters was performed, with at least 500 myotubes measured in each group. (**L**) Expression of MyHC, Atrogin-1, MuRF-1, RAC1, and SELENOW in the mRNA level of these primary myotubes; GAPDH was used as the loading control, *n* = 5 to 6. Data are means ± SEM; Student’s *t* test, *P ≤ 0.05, **P ≤ 0.01; labeled means without a common letter differ; *P* < 0.05.

Given the importance of mTOR signaling in proteostasis (*40*) and the crucial roles of RAC1 (*41*) and CDC42 (*42*) in this signaling, we also examined the protein level of RAC1 and CDC42 in GAS muscle. Here, we found that *SELENOW* KO notably decreased the protein expression of CDC42 in DEX-induced sarcopenia, whereas RAC1 was down-regulated in two sarcopenia models (Fig. 6, A to C). In addition, *SELENOW* KO down-regulated the phosphorylation of mTOR at S2248 in two sarcopenia models (Fig. 6, A to C). Subsequently, we examined the downstream protein level of mTOR associated with proteostasis. The activation of the mTORC1 pathway is inhibited in KO muscle, as illustrated by the down-regulation of EFI4G and phosphorylations of 4EBP1(Thr^45^) and -P70S6K (Ser^434^) (Fig. 6, D to F). Meanwhile, the activation of mTORC2-AKT-FOXOs was also inhibited in KO muscle, as shown by the down-regulated phosphorylations of AKT (Ser^473^) and FOXO3 (Ser^253^) (Fig. 6, G to I). To determine whether RAC1-mTOR activation is the direct mechanism through which SELENOW mediates proteostasis in skeletal muscle, we generated RAC1-knockdown primary myoblasts. Despite the fact that the loss of SELENOW appears to delay the differentiation of primary myoblasts into myotubes, MyHC staining confirms myotube differentiation and was used to assess myotube diameter (Fig. 6J). SELENOW overexpression mitigated DEX-induced primary myotube atrophy, whereas SELENOW overexpression with RAC1 knockdown failed to mitigate muscle atrophy (Fig. 6K). Compared with the vehicle group, the mRNA level of *MyHC* is down-regulated and *Atrogin-1* and *MuRF-1* are up-regulated in the DEX and DEX + SELENOW + siRAC1 group, whereas DEX + SELENOW shows intermediate mRNA levels between the vehicle and DEX group (Fig. 6L). Overall, the *SELENOW* KO-induced imbalance of proteostasis via the function of RAC1 in mTOR signaling.

### Overexpression of SELENOW alleviates DEX-induced myotube atrophy in primary myoblasts and C2C12 cells

**Fig. 7.**
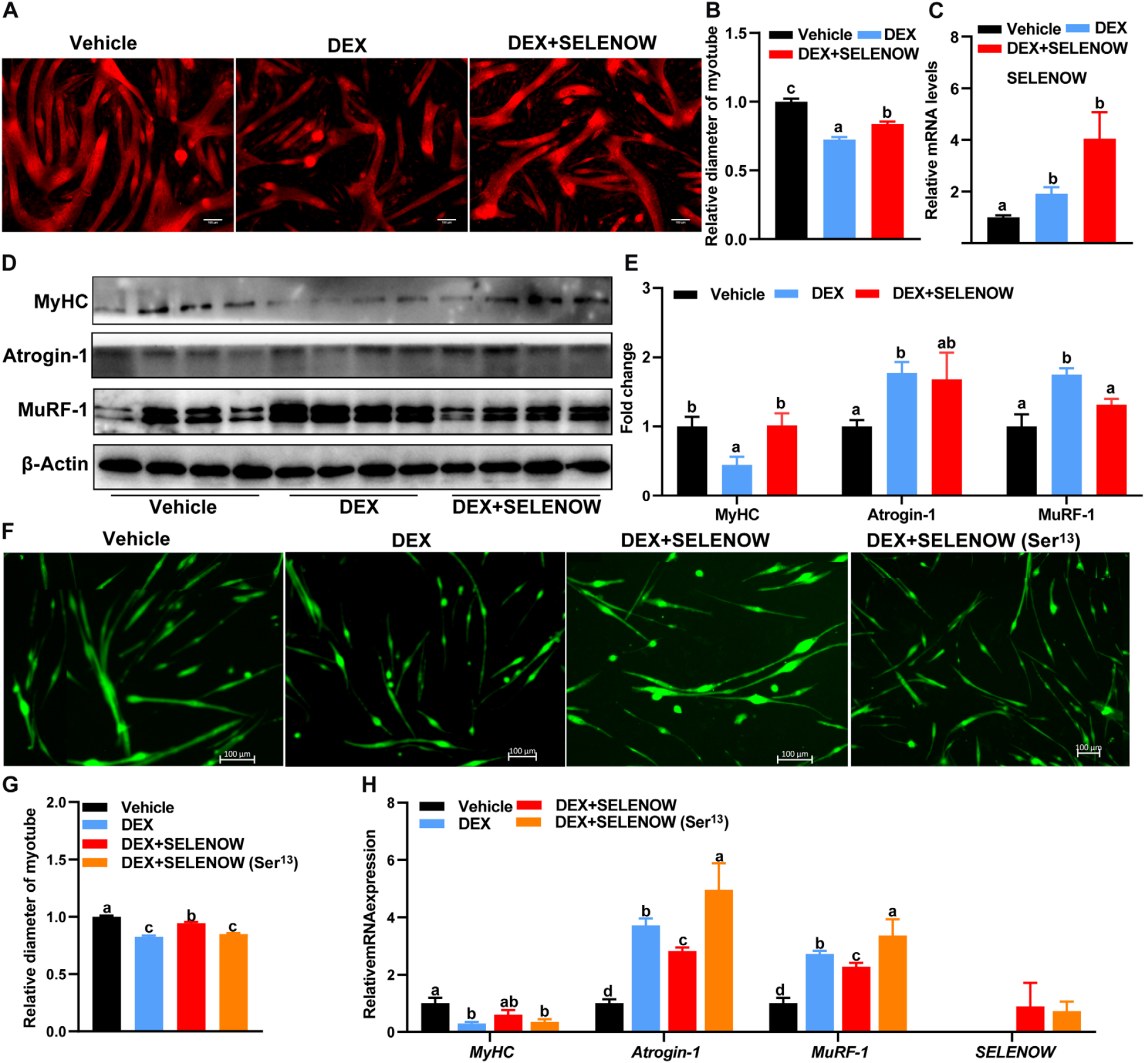
Overexpression of SELENOW alleviates myotube atrophy in vitro. (**A**) Immunofluorescent staining of MyHC in C2C12 myotubes. Before DEX treatment, the myotubes were infected with a control vehicle or a SELENOW vehicle; scale bars, 100 μm. (**B**) Quantification of these C2C12 myotube; at least 150 myotubes in one group were measured. (**C**) Expression of SELENOW in mRNA level of C2C12 myotube, *n* = 4. (**D** and **E**) Western blot analysis for the protein levels of MyHC, Atroging-1, and MuRF-1 in these C2C12 myotubes; β-actin was used as the loading control, *n* = 4. (**F**) Immunofluorescent staining of MyHC in primary myotubes. Before DEX treatment, the primary myotubes were infected with a control vehicle, a SELENOW vehicle, or a SELENOW (Ser^13^) vehicle; scale bars, 100 μm. (**G**) Quantification of these primary myotube diameters; at least 500 myotubes in one group were measured. (**H**) Expression of MyHC, Atrogin-1, MuRF-1, and SELENOW in the mRNA level of these primary myotubes; GAPDH was used as the loading control, *n* = 5 to 6. Data are means ± SEM; labeled means without a common letter differ; *P* < 0.05.

On the basis of the observation that *SELENOW* KO exacerbated muscle wasting in sarcopenia mice, we next examined whether high expression of SELENOW could rescue muscle atrophy in vitro. As compared with the vehicle group, we found that SELENOW could rescue muscle atrophy, as illustrated by the longer myotube diameter as compared with the DEX treatment group (Fig. 7, A to C). Accordingly, overexpression of SELENOW notably up-regulated the protein level of MyHC, whereas it down-regulated the level of MuRF1-1 (Fig. 7, D and E). In addition, SELENOW overexpression also mitigated DEX-induced primary myotube atrophy, whereas the recombinant SELENOW in which Sec^13^ was changed to Ser failed to mitigate myotube atrophy (Fig. 7, F and G). Compared with the vehicle group, the mRNA levels of *MyHC* are down-regulated and *Atrogin-1* and *MuRF-1* are up-regulated in the DEX and DEX + SELENOW (Ser^13^) group, whereas DEX + SELENOW shows intermediate mRNA levels between the vehicle and DEX group (Fig. 7H). These results indicated that the overexpression of SELENOW could alleviate myotube atrophy caused by DEX in vivo and the protective action depending on its selenocysteines.

### Overexpression of SELENOW alleviates DEX-induced muscle atrophy in mice

**Fig. 8.**
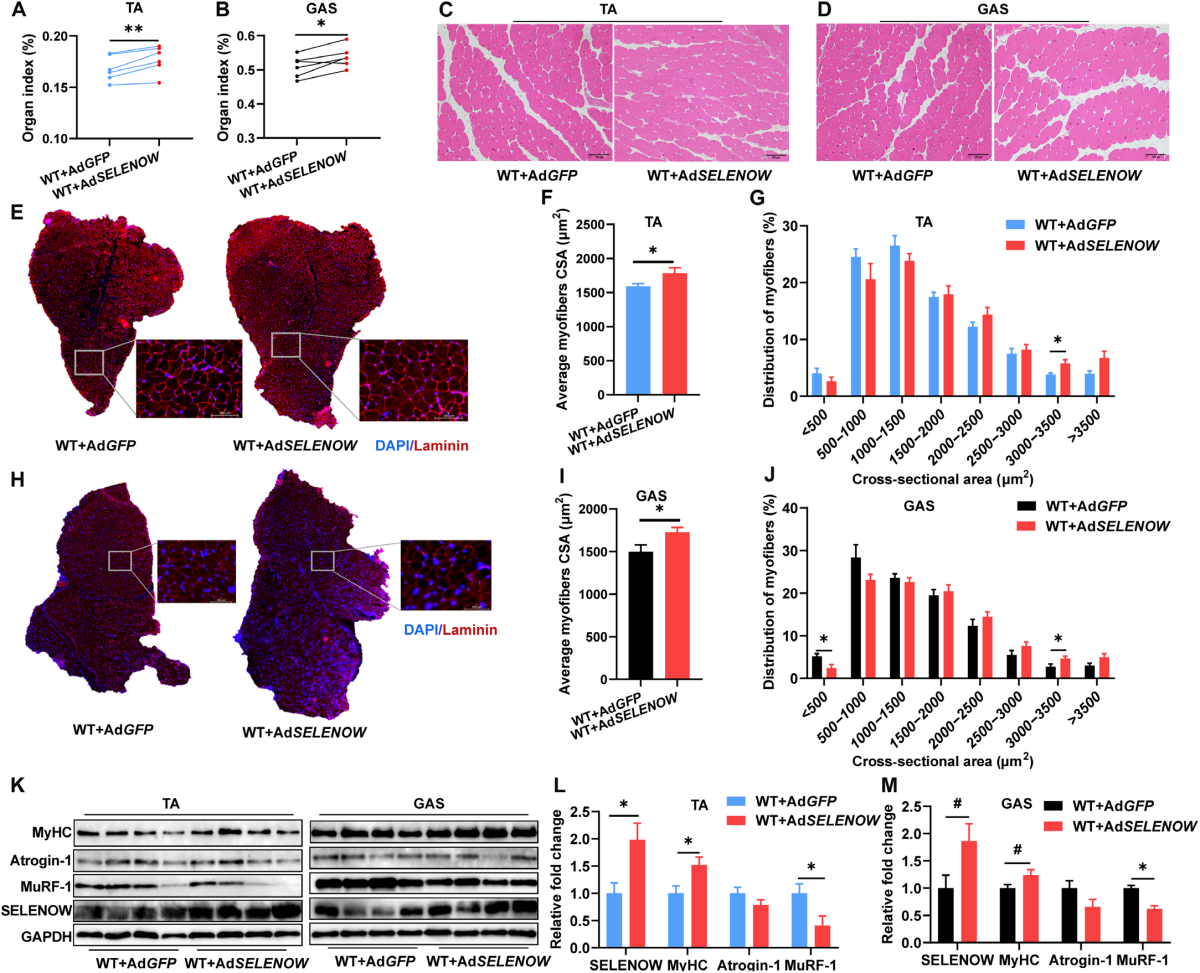
Overexpression of SELENOW alleviates muscle atrophy caused by DEX. (**A** and **B**) Mass analysis of TA and GAS in mice after adenovirus (Ad) injection with DEX treatment, *n* = 6. (**C** and **D**) Representative H&E staining of TA and GAS. (**E**) Laminin staining of myofiber cross section of TA muscle; scale bars, 100 μm. (**F** and **G**) Related myofiber CSA and frequency of distribution for CSA of TA muscle, *n* = 6. (**H**) Laminin staining of myofiber cross section of GAS muscle; scale bars, 100 μm. (**I** and **J**) Related CSA and frequency of distribution for CSA of GAS muscle a, *n* = 6. (**K** to **M**) Western blot analysis of protein levels of MyHC, Atrogin-1, and MuRF-1 in TA and GAS muscle. GAPDH was used as the loading control, *n* = 4. Data are means ± SEM; Student’s *t* test and paired *t* test were used in (A) and (B); unpaired *t* test was used for the others, *P ≤ 0.05, **P ≤ 0.01, ^#^*P* ≤ 0.1.

To further determine the effects of SELENOW on muscle mass and function in vivo, a SELENOW overexpression vector of adenovirus (Ad) was constructed. Two-month-old male mice were treated with DEX for 10 days via intraperitoneal injections of 25 mg kg^−1^ day^−1^, and adenovirus was injected twice (on day 4 and day 5 from the first DEX injection) into the TA and GAS muscles. Compared with Ad-*GFP* mice, the weights of TA and GAS muscles were up-regulated in Ad-*SELENOW* mice (Fig. 8, A and B). No pathological change was observed in TA and GAS muscle between Ad-*GFP* and Ad-*SELENOW* mice (Fig. 8, C and D). Accordingly, Ad-*SELENOW* mice had higher mean CSA of individual myofibers in TA muscles (Fig. 8, E to G) and similarly in GAS muscles (Fig. 8, H to J). In addition, MuRF-1 was down-regulated in TA and GAS muscles, whereas SELENOW and MyHC were up-regulated in TA or GAS muscles of Ad-*SELENOW* mice (Fig. 8, K to M). Altogether, these results further demonstrated that overexpression of SELENOW alleviates mice muscle atrophy in the condition of muscle atrophy.

## DISCUSSION

Here, we found that the expression level of SELENOW was up-regulated under muscle atrophy and sarcopenia conditions. We provided compelling physiological, histological, and molecular evidence to demonstrate that ablation of SELENOW profoundly aggravates the process of muscle mass loss by regulating protein synthesis and degradation in mouse models of muscle atrophy and sarcopenia. In addition, we found that overexpression of SELENOW in vivo and in vitro alleviated the muscle atrophy induced by DEX. These findings provide a fresh insight into the association between Se deficiency and muscle disease and provide promising evidence for the prevention and treatment of sarcopenia. We suggest the crucial role of SELENOW in skeletal muscle during aging.

Sarcopenia can have characteristics such as skeletal muscle mass loss and fiber atrophy, which might appear acutely (usually in the setting of acute diseases) or chronically (mainly are aging) (*43*). Because the early pathology symptoms of sarcopenia are not obvious and are easy to ignore in patients, animal models are being used to understand the pathophysiology of sarcopenia (*44*). Here, we established a DEX-induced acute muscle atrophy and a naturally chronically occurring aging-associated sarcopenia according to previous studies (*44*, *45*). We found that *SELENOW* KO aggravated muscle loss in both DEX-induced muscle atrophy and aging-associated sarcopenia mice models, whereas there was no influence on muscle mass in the basal condition. Of note, *SELENOW* KO mainly affects type IIA fibers in TA muscle of sarcopenia mice. The loss of SELENOW may progressively exacerbate muscle atrophy as age increases, supported by the decreasing size of type IIA fibers in muscle during aging (*38*). Here, we also noticed that physiological levels of SELENOW in the normal muscle are not enough to prevent muscle atrophy or contribute to muscle growth. A previous study has indicated that only minor defects in muscle morphology were observed in SELENON KO mice, whereas the obvious phenotype was observed under stress conditions (forced swimming test) (*46*). In addition, a recent study demonstrated that overexpression of another selenoprotein, GPX4, could mitigate muscle mass loss in aged mice, while not affecting muscle mass in younger mice (*47*). These results reinforce the concept that standard selenium levels and its associated selenoproteins are optimized for everyday metabolic needs and typical oxidative stress encounters, where numerous studies have indicated the increased demand for selenium under some pathological conditions (*48*–*50*). In the face of stressors such as corticosteroids or the natural aging process, the increased oxidative and proteolytic stresses (*51*, *52*) might surpass these foundational safeguards, which induced physiological levels of SELENOW insufficient to prevent muscle atrophy. Correspondently, loss of SELENOW accelerates muscle atrophy under such conditions. Therefore, the SELENOW modulation in selenium deprivation or over-supplementation within the context of muscle atrophy is meaningful and requires further research in the future.

Previous studies have indicated that SELENOW plays an essential role in cellular redox by binding to GSH (*30*) and the loss of SELENOW has increased ROS levels in macrophages (*53*). The loss of SELENOW in sarcopenic mice increases cellular oxidative damage, evidenced by elevated MDA levels, a recognized marker of oxidative stress and lipid peroxidation (*54*). In addition, KEGG enrichment analysis of DEGs between WT and KO in TA muscle of sarcopenia mice also revealed the up-regulated ROS and glutathione metabolism. These results indicated that the loss of SELENOW aggravates muscle atrophy, which may be attributed to an oxidation imbalance.

The activation of Atrogin-1 and MuRF-1 is the common key regulatory step contributing to the protein degradation in atrophying muscle (*55*, *56*), whereas the imbalance between protein synthesis and degradation is a crucial cause of muscle mass loss in sarcopenia (*57*). In current studies, Atrogin-1 and MuRF-1 were up-regulated in the KO muscle of DEX-induced muscle atrophy and aging-associated sarcopenia mice. These results indicated aggravated protein loss in *SELENOW* KO muscle under atrophy conditions. Rho GTPase was reported to play a crucial role in skeletal muscle including muscle system development and muscle mass homeostatic balance (*58*). Here, GO enrichment analysis of DEGs between WT and KO in TA muscle of sarcopenia mice showed that *SELENOW* KO notably up-regulated the regulation of Rho protein signal transduction. Meanwhile, *SELENOW* KO down-regulated cytoplasmic translation and muscle system processes, which are closely related to protein synthesis and muscle homeostasis (*59*, *60*). Moreover, the GO enrichment analysis of the proteins interacting with SELENOW indicated that SELENOW is associated with proteostasis and GTPase signaling transduction. These results suggest that SELENOW functions in proteostasis via Rho protein signal transduction.

RAC1, a member of Rho GTPase, was reported to play a crucial role in mTOR signaling in both mTORC1 and mTORC2 and controls cellular size (*41*), while the kinase mTOR serves as a critical hub for protein production and degradation (*40*, *61*). A previous study showed the interaction between SELENOW and RAC1 by immunoprecipitation mass spectrometry (*39*). Here, we further demonstrated the protein interaction between SELEOW and RAC1 by co-IP, laser confocal microscopy, and protein binding mode prediction. Although there are two ionic bonds and seven hydrogen bonds between SELENOW and RAC1, the Sec residue may be the most critical. Three binding sites were identified at Sec^13^ of SELENOW, and the recombinant SELENOW, in which Sec^13^ was replaced with serine, failed to mitigate DEX-induced primary myotube atrophy. Despite the possibility that RAC1 interacts with SELENOW via intermediary bridging molecules because 14-3-3 proteins can bind to SELENOW (*62*) and RAC1 (*63*), there is no doubt that SELENOW may regulate RAC1 functions via protein interactions. Moreover, we found that *SELENOW* KO down-regulated the protein level of RAC1 in skeletal muscle of sarcopenia mice. In previous studies, SELENOW was reported to suppress EGFR ubiquitination (*64*) and could function as an adaptor to bridge TRIM21 to its binding proteins and then degraded these molecules in a proteasome-dependent manner (*39*), which implies that SELENOW could posttranslationally modify to its target protein. Here, we indicated that SELENOW may be involved in posttranslational modification of RAC1 via protein interaction.

Recent studies have shown that selenium deficiency mediates protein turnover by inhibiting mTOC1 signaling and stimulating the ubiquitin-proteasome pathway in skeletal muscle (*65*); however, which selenoproteins function in skeletal muscle proteostasis remains unclear. Here, we postulated a SELNOW-RAC1-mTOR cascade as a potential mechanism mediating protein synthesis and degradation in skeletal muscle. In the absence of RAC1, both mTORC1 and mTORC2 fail to phosphorylate their downstream targets (*41*). Protein synthesis is the central function of mTORC1, which signals the translation of machinery via the phosphorylates 4EBP1 and S6Ks (*66*, *67*), then S6Ks controls several proteins linked to translation, including ribosomal proteins S6 (*68*) and eIF4G (*69*). Here, we found that *SELENOW* KO down-regulated the protein levels of EIF4G, phosphorylation of 4EBP1 at Thr^45^, and phosphorylation of p70S6K at Ser^434^ in GAS muscle, which indicated that the translation process is suppressed in the KO group. The major protein degradation pathway in skeletal muscle is the ubiquitin-proteasome system (*70*). Transcription factors FOXOs drive atrophy-related ubiquitin ligase Atrogin-1 and MuRF-1 expression (*71*), and mTCRC2 is required for signaling to AKT-FOXOs (*72*). Previous research has established that SELENOW regulates mTORC2-AKT signaling (*73*). Here, we found that the phosphorylation of AKT at Ser^473^ and phosphorylation of FOXO3a at Ser^253^ are down-regulated in the KO group, which suggests an accelerated protein degradation process. Moreover, overexpression of SELENOW failed to alleviate DEX-induced primary myotube atrophy in RAC1-knockdown myoblasts, indicating that SELENOW is directly involved in the RAC1-mTOR activation. Collectively, we indicated that SELENOW could regulate protein synthesis and degradation by the SELENOW-RAC1-mTOR cascade and explain selenium’s function in proteostasis.

Here, we found that the overexpression of SELENOW could alleviate the muscle atrophy caused by DEX in vivo and in vitro. Meanwhile, overexpression of SELENOW regulated the atrophy biomarkers (MuRF-1 and MyHC), which further verify SELENOW’s function in proteostasis. In addition, previous studies have indicated that selenium supplementation is an important strategy to prevent muscle disorders or improve skeletal muscle performance (*65*, *74*, *75*), and the increased protein synthesis is accompanied by the up-regulation of SELENOW in skeletal muscle (*76*). These results underscore the pivotal role of SELENOW in muscle atrophy and imply the potential in the selenium-mediated prevention of muscle disorders.

Nevertheless, we found that loss of SELENOW appears to delay the differentiation of primary myoblasts into myotubes compared with standardized primary myoblast differentiation (*77*). These outcomes are similar to a previous study that showed suppressed myogenic differentiation in SELENOW-knockdown C2C12 cells (*35*). Similar results were also found in another selenoprotein, primary myoblasts from GPX1 null mouse showing a defect in myogenic differentiation (*78*). In addition, selenium supplementation could alleviate the negative effect of heat stress on myogenic differentiation of C2C12 cells with the response of selenogenome (*79*). These studies imply the crucial role of selenium and selenogenome in satellite cells. Given their high potential to proliferate and differentiate into muscle fibers, satellite cells are widely recognized for their contributions to the maintenance of muscle mass and hypertrophy (*80*), and the function of SELENOW in maintaining muscle mass may also be through the regulation of satellite cells.

In conclusion, our study indicates that SELENOW is the age-related selenoprotein gene and is important for skeletal muscle protein homeostasis, which provides valuable insights into the relationship between selenium and sarcopenia. Beyond their relevance for understanding the mechanism of sarcopenia, these findings have potential implications for the prevention and treatment of sarcopenia.

### Limitations of the work

Our work revealed the crucial role of SELENOW in the regulation of proteostasis in skeletal muscle. However, the function of SELENOW has not been verified in other muscle atrophy models, such as starvation- or denervation-induced muscle atrophy. In addition, the mechanistic role of selenium-SELENOW in muscle with the aging-related sarcopenia or DEX-induced muscle atrophy mice under selenium deprivation or over-supplementation conditions has yet to be explored. Nonetheless, our results provide compelling evidence for the function of SELENOW in sarcopenia.

## MATERIALS AND METHODS

### Mice

All animal experiments were performed in accordance with the National Research Council Guide for the Care and Use of Laboratory Animals and approved by the Institutional Animal Care and Use Committee at Huazhong Agricultural University (HZAUMO-2019-102). WT and SELENOW-KO mouse lines were maintained on a C57BL/6N background. Mice were caged in groups (five per cage) located in a room at 22° ± 2°C and with a 12-hour light/dark cycle, given free access to diet and water, and checked daily. If not stated differently, young male mice were 2 to 3 months old and aged male mice were 22 to 24 months old. For DEX-induced muscle atrophy, 2-month-old male mice were treated with DEX for 8 to 10 days via intraperitoneal injections of 25 mg kg^−1^ day^−1^, as was previously done (*37*). For adenovirus injection, a total of 1 × 10^9^ PFU and 2 × 10^9^ PFU SELENOW-expressing adenoviruses were intramuscularly injected into five to six sites of the TA and GAS muscles in 2-month-old male mice on day 4 and day 5 from the first DEX injection, respectively. In the analysis of age-related sarcopenia, young mice were raised until 22 to 24 months old, as previously reported (*81*).

### Isolation of primary myoblasts

The primary myoblasts were isolated as previously described (*77*). Briefly, hindlimb muscles from 6- to 8-week-old mice were minced into a coarse slurry using razor blades and enzymatically digested at 37°C in 10 ml of collagenase II (Worthington Biochemical) solution at a concentration of 400 U/ml. After 1 hour of digestion, the slurry was passed through a 70-μm and then a 30-μm cell strainer (Miltenyi Biotec). The filtrate was centrifuged at 1400*g* for 5 min at room temperature to sediment the dissociated cells. The pellet was resuspended and grown in Ham’s F-10 nutrient mixture (Thermo), containing penicillin (200 U/ml) and streptomycin (200 μg/ml), and supplemented with 20% fetal bovine serum (FBS; Gibco) and basic FGF (5 ng/ml; PeproTech Inc., Rocky Hill, NJ). Cellular mixtures were seeded onto a 10% Matrigel (Corning)–precoated dish. After culture until cells were sufficient, the cells were detached with trypsin, resuspended in growth media, and transferred to a non–Matrigel-coated tissue culture dish for 45 min. Subsequently, all the supernatant was transferred to a new Matrigel-coated dish. Repeat the cell purification steps above until a myoblast purity of >98% is achieved.

### Cell culture

C2C12 cell lines were grown in Dulbecco’s modified Eagle’s medium (DMEM), supplemented with 5% heat-inactivated FBS, penicillin (100 U/ml), streptomycin (100 mg/ml), 1 mM sodium pyruvate, and 10 mM Hepes at 37°C in 5% CO_2_ at 100% humidity. To induce differentiation, cells were planted on culture medium coated with 0.1% Matrigel, and when cell confluence reached 70%, the medium was switched into differentiation medium (DMEM containing 2% horse serum) for 4 to 6 days and multinuclear myotubes were formed. To induce myotube atrophy, cells were incubated with 50 mM DEX in DMEM for 24 hours.

### Transfection

The sequences of SELENOW, containing CDS and the SECIS, were generated by PCR using cDNA extracted from murine muscle and then cloned into pcDNA 3.1+ plasmid. A point mutation in mouse of SELENOW in which Sec^13^ was changed to Ser^13^ was generated by site-directed mutagenesis of the cDNA through PCR using the pcDNA 3.1+/ SELENOW plasmid. Two siRNA duplexes (siRNA1: 5′-GCCTAGACATTCAAGACAA-3′; siRNA2: 5′-AGACGGAGCCGUUGGUAAATT-3′) that target the coding region of RAC1 were used. Differentiation medium was replaced with Opti-MEM media (Gibco) when multinuclear myotubes were formed. pcDNA3.1-SELENOW, pcDNA3.1-SELENOW (Ser^13^) plasmid, or siRNA-RAC1 was transfected into myotubes by Lipofectamine 2000 reagent according to the manufacturer’s instructions. Opti-MEM media was replaced with DMEM after 6 hours and cells were cultured for 24 hours after transfection and fixed or lysed for further analysis.

### Grip strength and hanging time test

The grip strength test and the hanging time test were performed as previously described (*81*). For the grip strength test, mice were trained to grasp a grid attached to an electronic dynamometer (Sanliang SMF-20N, China). During testing, we pulled the mice gently backward, parallel to the dynamometer, until they released the bar. The force applied was recorded as the grip strength. Each mouse underwent four tests spaced 1 week apart, with six trials per test, and the average results were recorded. In the hanging time test, a 45 cm × 45 cm grid with 2-mm bar thickness and 18-mm mesh was used, mounted 50 cm above a 5-cm-thick cushion. Mice were placed at the grid center, which was then flipped upside down. Hanging time lasted until the mice fell. Each mouse underwent four tests spaced 1 week apart, and the average hanging time was recorded.

### Glucose and insulin tolerance testing

Before the studies, mice were fasted overnight for glucose tolerance testing (GTT) or for 4 hours for insulin tolerance testing (ITT). GTT and ITT were performed as described previously (*82*). In the GTT, d-glucose was administered at 1.5 g/kg body weight, whereas for the ITT, a 0.75 U/kg dose of human regular insulin (Sigma-Aldrich) was administered intraperitoneally. Blood glucose concentrations were subsequently measured at intervals of 0, 15, 30, 60, 90, and 120 min postchallenge using a glucose meter (Sinocare, China).

### Redox analysis

Muscle MDA and GSH concentrations were determined using specific assay kits (A003-1-2 and A005-1-2; Nanjing Jiancheng Bioengineering Institute). TXNRD activity was determined with a specific assay kit (TRXR-1-W; Suzhou Comin Biotechnology Co. Ltd.). The concentration of GPX4 in mouse tissue was determined with a specific assay kit (JN20546; Shanghai Jining Shiye Co. Ltd).

### Histological and immunofluorescence assessment

TA muscle from WT and KO mice was bisected at the mid-belly and frozen in an OCT compound then cross-sectioned at 6 μm thickness using a Leica CM3050SL cryostat. Slides were subjected to histological staining or immunofluorescence staining. H&E staining of muscle sections was performed according to a previously reported method (*83*). Briefly, the slides were first stained in hematoxylin for 15 min, rinsed in running tap water for 20 to 30 min and then stained in eosin for 1 to 2 min. Slides were dehydrated in graded ethanol and xylene, then mounted in Permount (Thermo Fisher Scientific). Slides were visualized using an optical microscope (BX53; Olympus, Japan).

Immunofluorescence staining was performed as described previously (*84*). Briefly, cultured cells or cross sections were fixed in 4% paraformaldehyde (PFA) in phosphate-buffered saline (PBS) for 10 min, quenched with 100 nM glycine for 10 min, and incubated in blocking buffer (5% goat serum, 2% bovine serum albumin, 0.1% Triton X-100, and 0.1% sodium azide in PBS) for at least 1 hour at room temperature. Samples were then incubated with primary antibodies diluted in blocking buffer (dilution ratio as shown in table S2) overnight at 4°C. After washing with PBS, samples were incubated with secondary antibodies and DAPI (4′,6-diamidino-2-phenylindole) for 1 hour at room temperature. Samples were visualized using a fluorescence microscope (DMi8; Leica, Germany) or a confocal laser scanning microscope (LSM800; Zeiss, Germany).

### Fiber cross-sectional and myotube diameter analysis

The CSAs of fibers in TA muscle were analyzed by SMASH software based on the myofiber borders identified by laminin immunostaining; at least 200 fibers per sample were measured. All fibers in the cross-sectional images were quantified unless the sarcolemma was not intact. The diameters of myotubes were analyzed by ImageJ based on MyHC immunostaining. Three different sites in each myotube were measured, with a minimum of 150 myotubes from C212 and 500 from primary myoblasts per group.

### Real-time PCR and RNA-seq

Total RNA was extracted from the muscle using RNA purification kits (Takara, cat no. 740984.50) according to the manufacturer’s instructions. For real-time PCR, 1 μg of total RNA was reverse-transcribed using random primers with ABScript III RT Master Mix for qPCR (ABclonal, cat no. RK20429). Real-time PCR was carried out in a Bio-Rad CFX384 system (Bio-Rad) with 2X Universal SYBR Green Fast qPCR Mix (ABclonal, cat no. RK21203) and gene-specific primers that are listed in table S3. The 2^−ΔΔCt^ method was used to analyze the relative changes in each gene’s expression normalized against glyceraldehyde-3-phosphate dehydrogenase (GAPDH) mRNA expression.

The RNA-seq analysis was performed as previously described (*85*). Briefly, three RNA samples of muscle from each group were prepared and submitted to Wuhan Seqhealth Technology Co. Ltd. (Wuhan, China). After RNA quality assessment (RNA integrity number > 7.5), sequencing was performed on an Illumina Hiseq platform and 150-bp paired-end reads were generated. Reads of each sample were aligned to the GRCm38 using Hisat2 (v2.1.0). Feature Counts (v1.6) was used to count the read numbers mapped to each gene. DEGs were identified with the DESeq2 R package (1.32.0) using a *P* value <0.05 and fold change >1.5 or <0.7. GO and KEGG enrichment analyses of DEGs were implemented by the Cluster Profiler (4.0.2) R package and the Benjamini and Hochberg FDR multiple testing correction with FDR < 0.05. The heatmap of DEGs was illustrated by TBtools.

### Western blotting

Total proteins were extracted from muscle tissue and cells using radioimmunoprecipitation assay (RIPA) buffer with 1% (v/v) phenylmethylsulfonyl fluoride (Beyotime Biotechnology, cat no. P0013B). Protein concentrations were determined using Pierce BCA Protein Assay Reagent (Beyotime Biotechnology, cat no. P0009). Proteins were separated by SDS-PAGE, transferred to a polyvinylidene difluoride membrane, blocked in 5% fat-free milk for 2 hours at room temperature, and then incubated with primary antibodies in 3% bovine serum albumin overnight at 4°C. The membrane was then incubated with secondary antibody for 1 hour at room temperature. The primary antibodies used for the analyses are presented in table S3. Immunodetection was performed using enhanced Western Blotting Chemiluminescence Luminol Reagent and detected with a Chemi-Image System (Tanon5200, China). All protein levels were normalized to that of the housekeeping protein GAPDH or β-actin, and densitometry quantification of the Western blotting bands was performed using ImageJ software.

### Immunoprecipitation

Immunoprecipitation procedures were performed as previously described (*73*). Briefly, lysates were mixed with antibodies overnight, and the immune complexes were then incubated with protein A or G beads for 1.5 hours. The beads were washed and boiled with 2× SDS sample buffer for 3 min. The samples were loaded onto SDS-PAGE gels, transferred to a membrane, and then incubated with primary antibody at 4°C overnight. After further incubation with horseradish peroxidase–conjugated secondary antibody for 1 hour at room temperature, immunoreactive bands were visualized using a West Pico enhanced ECL detection kit (Pierce), and the intensity was determined densitometrically using ImageJ software.

### Molecular docking

The binding affinity between RAC1 and SELENOW was confirmed using a molecular docking approach. The tertiary structures of target proteins were obtained from the Protein Data Bank database (http://wwpdb.org/). Molecular docking was performed using the ClusPro Server (https://cluspro.bu.edu/home.php). Interaction interface residue analysis was performed using the software PyMOL and Ligplot.

### Statistical analysis

If not stated differently, besides the RNA-seq data, the other data were analyzed by Student’s *t* test with Welch’s correction for unequal variances. Data are presented as means ± SEM, *P* < 0.05 was considered as statistically significant. The statistical analysis and image presentation were performed in GraphPad Prism, version 9.0.
