# Supplementary material for: Loss of SELENOW aggravates muscle loss with regulation of protein synthesis and the ubiquitin-proteasome system
Source: Sci Adv. 2024 Sep 20;10(38):eadj4122. doi: 10.1126/sciadv.adj4122 (PMC11414739; doi:10.1126/sciadv.adj4122)
Supplement: Supplementary file 1 — Supplementary Text Figs. S1 to S5 Tables S1 to S3 Legend for table S4 [file sciadv.adj4122_sm.pdf]

Science Advances
AAAS

Supplementary Materials for

Loss of SELENOW aggravates muscle loss with regulation of protein synthesis and the ubiquitin-proteasome system

Jia-Cheng Yang *et al.*

Corresponding author: Lv-Hui Sun, lvhuisun@mail.hzau.edu.cn

Sci. Adv. **10**, eadj4122 (2024)
DOI: 10.1126/sciadv.adj4122

The PDF file includes:

Supplementary Text
Figs. S1 to S5
Tables S1 to S3
Legend for table S4

Other Supplementary Material for this manuscript includes the following:

Table S4

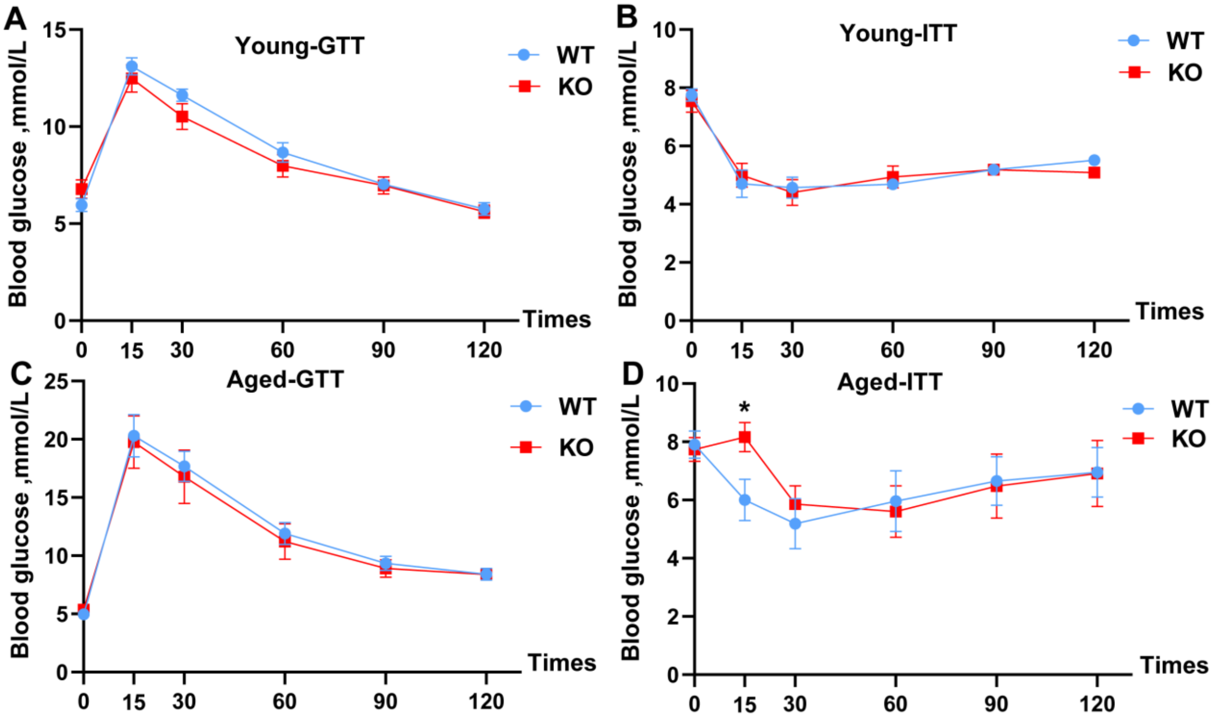

Fig. S1 Glucose tolerance tests (GTT) and insulin tolerance tests (ITT) in wild type and KO young and aged mice.

(A-B) GTT and ITT results of 2-month old mice, n=6-7

(C-D) GTT and ITT results of 24-month old mice, n=8

Intraperitoneal glucose tolerance and insulin tolerance test was performed, and plasma glucose levels were measured at 0, 15, 30, 60, and 120 min. Data are means ±SEM; Student's t test, *$P \leq 0.05$.

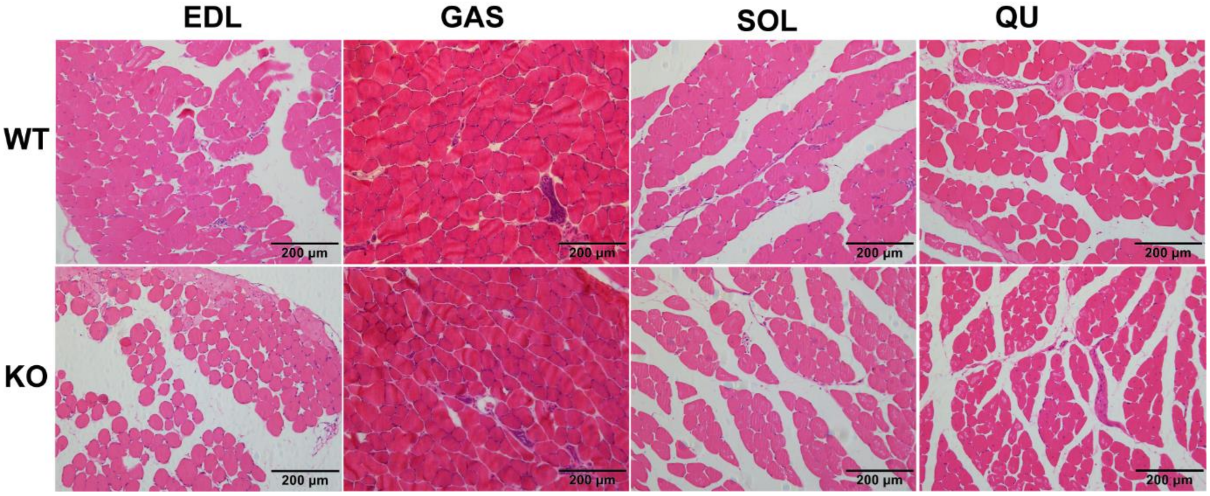

Fig. S2 Effect of *SELENOW* KO on morphology of different muscle tissues in aged mice

Representative H&E staining of EDL, GAS, SOL, and QU muscle, n=3-4, scar bar=200 μm.

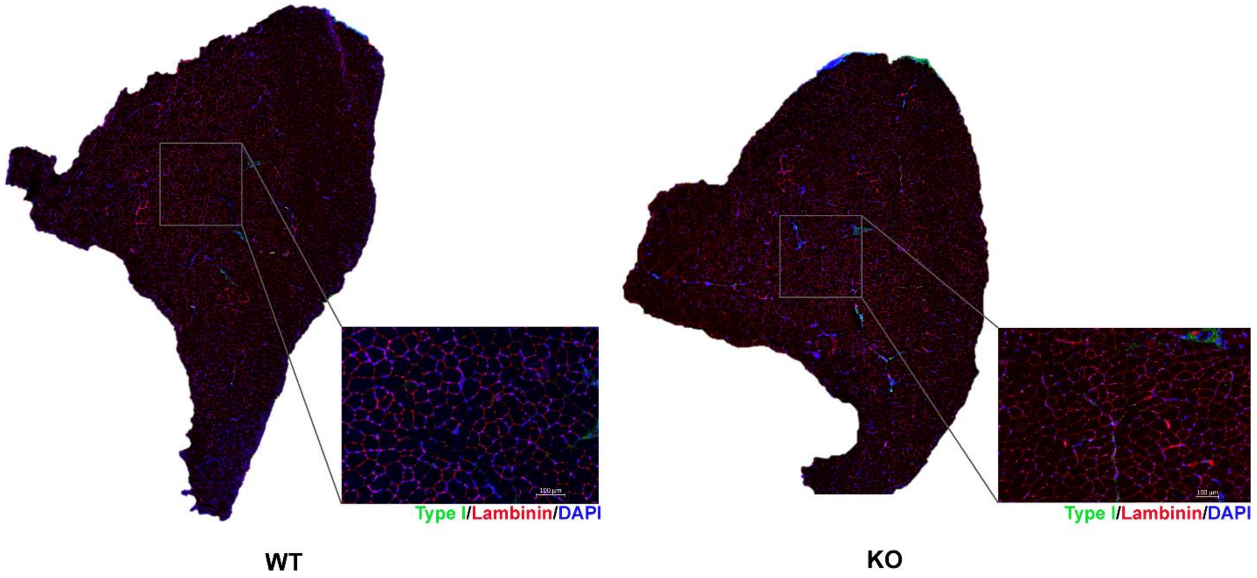

Fig. S3 Effect of *SELENOW* KO on Type I fibers in aged mice.

Representative Laminin staining of Type I fibers of TA muscl**e**, n=5, scale bar=100 μm.

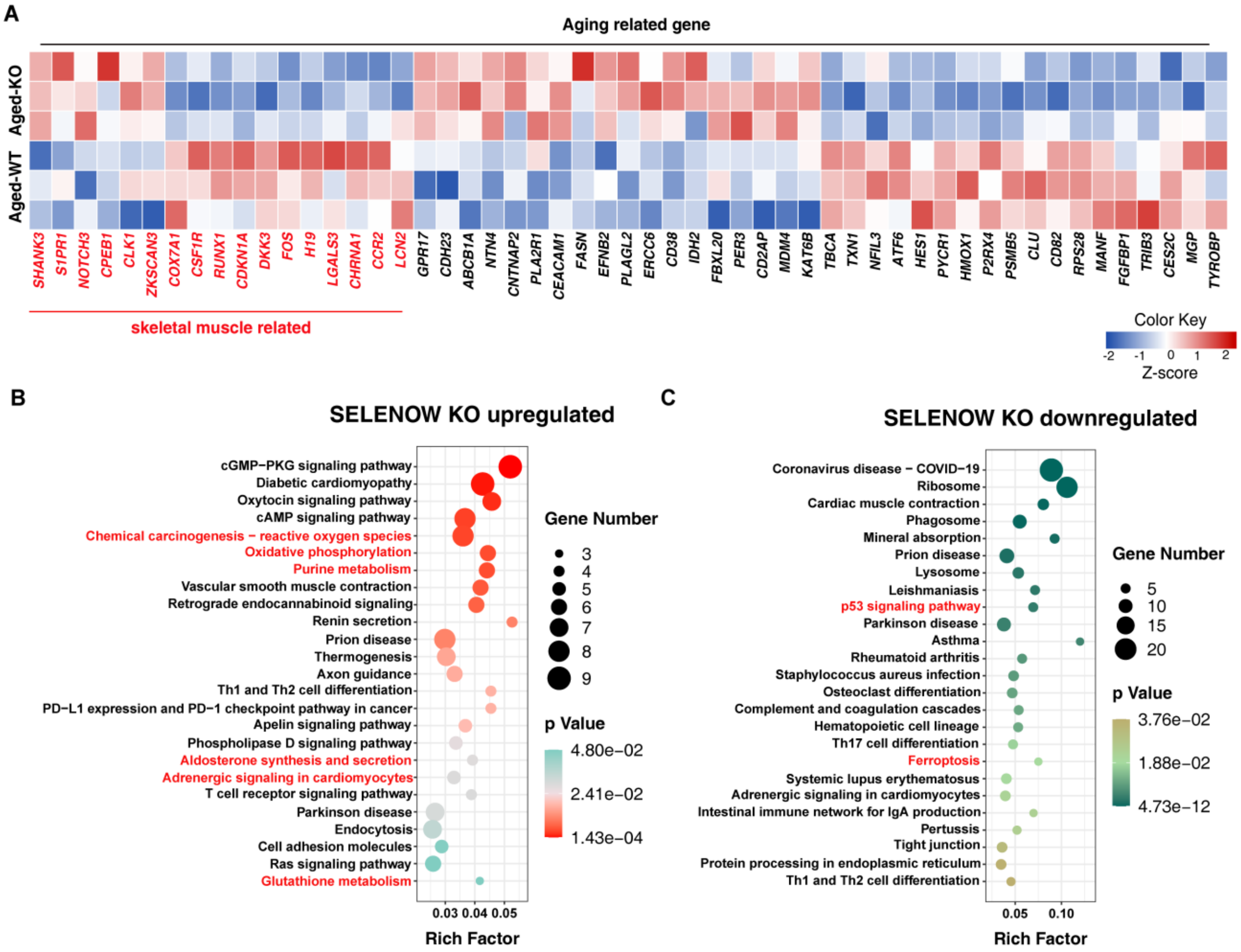

Fig. S4. KEGG pathway enrichments and heat maps in aged mice between WT and KO.

(A) Heat map diagram showing the downregulated and upregulated genes related to aging in TA muscle of SELENOW KO versus WT mice, genes marked in red font also relate to skeletal muscle homeostasis, n=3.

(B-C) KEGG analysis showing the enrichment of upregulated and downregulated pathways in TA muscle of SELENOW KO versus WT mice, the pathways marked in red font are associated with ROS metabolic process, n=3.

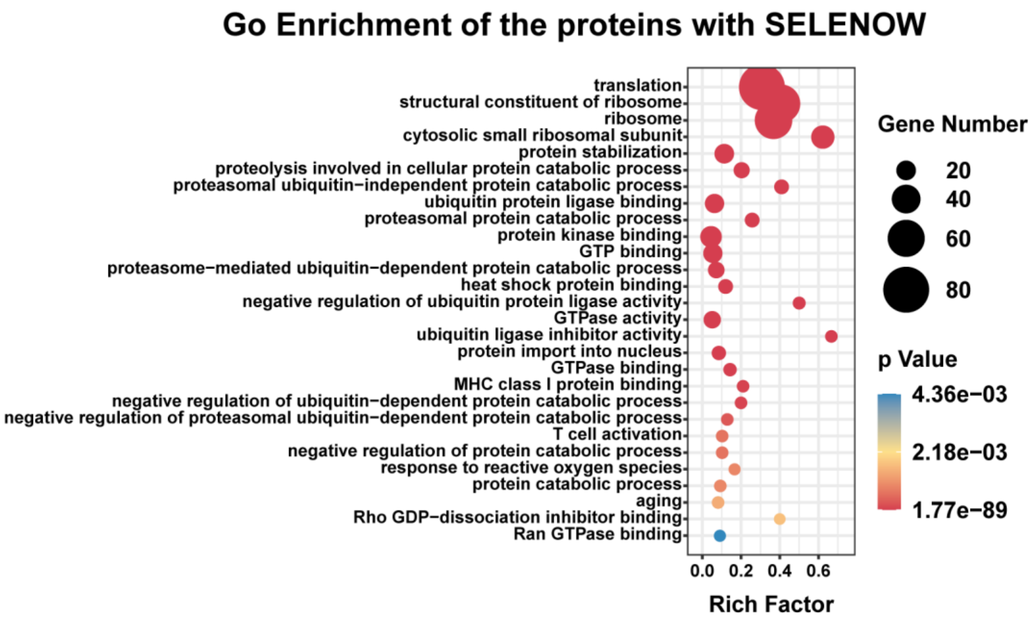

Fig. S5 GO enrichments of the proteins interact with SELENOW.

Top 30 pathways associated with proteostasis and GTPase signalling transduction.

Table S1. DEGs associate with aging and supporting paper sources

Gene name	Reference	Gene name	Reference	Gene name	Reference
CDKN1A	PMID: 34035185	PSMB5	PMID: 30816680	MDM4	PMID: 33688918
CSF1R	PMID: 34867307	ATF6	PMID: 29423270	FBXL20	PMID: 32898767
DKK3	PMID: 29717119	CLU	PMID: 22795969	GPR27	PMID: 33675110
CLK1	PMID: 16889924	MGP	PMID: 12871556	NTN4	PMID: 30514230
H19	PMID: 30107531	TYROBP	PMID: 30548312	ERRC6	PMID: 16754848
RUNX1	PMID: 26002684	CES2C	PMID: 22446518	KTA6B	PMID: 32014431
LCN2	PMID: 34325073	CD82	PMID: 32088828	EFNB2	PMID: 26980243
ZKSCAN3	PMID: 32427330	TXN1	PMID: 21873593	PER3	PMID: 17309758
SHANK3	PMID: 36436456	HMOX1	PMID: 30009872	PLA2R1	PMID: 30216637
COX7A1	PMID: 18488190	TBCA	PMID: 8544776	CEACAM1	PMID: 31389127
CCR2	PMID: 31843499	FGFBP1	PMID: 28053031	CDH23	PMID: 33179774
LGALS3	PMID: 30886760	PYCR1	PMID: 31091804	IDH2	PMID: 29567975
FOS	PMID: 36840360	HES1	PMID: 33561419	CD38	PMID: 35687485
SLPR1	PMID: 32544390	TRIBP3	PMID: 33532126	CNTNAP2	PMID: 25139204
NOTHC3	PMID: 31220525	P2RX4	PMID: 23303206	PLAGL2	PMID: 34916302
CPEB1	PMID: 36697412	NFIL3	PMID: 23209438	FASN	PMID: 30962418
RPS28	PMID: 33974070	ABCB1A	PMID: 19429129		
MANF	PMID: 31489403	CD2AP	PMID: 31440393		

Note: PMID is the PubMed reference number.

Table S2 Name, type, dilution, and source of primary antibodies

Antibody	Source	IDENTIFIER
Rabbit monoclonal anti-4EBP1	HuaBio	Cat#ET1701-83
Rabbit monoclonal anti-Phospho-4EBP1-T45	HuaBio	Cat#RT1004
Rabbit polyclonal anti-AKT	Abclonal	Cat#A11016; RRID:AB_2758365
Rabbit monoclonal anti-Phospho-AKT-S473	Cell signaling technology	Cat#4060; RRID:AB_2315049
Mouse monoclonal anti-Atrogin-1	Santa Cruz	Cat#sc-166806; RRID:AB_2246982
Rabbit polyclonal anti-CDC42	Abclonal	Cat#A19028; RRID:AB_2862520
Rabbit polyclonal anti-EIF4G	HuaBio	Cat#ER63337
Rabbit polyclonal anti-FOXO3	Abclonal	Cat#A0102; RRID:AB_2756951
Rabbit monoclonal anti-Phospho-FOXO3-S253	HuaBio	Cat# ET1609-49, RRID:AB_3069859
Rabbit polyclonal anti-GAPDH	Abclonal	Cat#AC001; RRID:AB_2619673
Rabbit monoclonal anti-mTOR	Cell signaling technology	Cat#2983; RRID:AB_2105622
Rabbit polyclonal anti-Phospho-mTOR-S2448	Abclonal	Cat#AP0094; RRID:AB_2771340
Mouse monoclonal anti-MuRF-1	Santa Cruz	Cat#sc-398608; RRID:AB_2819249
Mouse monoclonal anti-MyHC	Santa Cruz	Cat#sc-376157; RRID:AB_10989398
Rabbit polyclonal anti-P70S6K	Abclonal	Cat#A2190; RRID:AB_2749844
Mouse monoclonal anti- Phospho -P70S6K-S434	HuaBio	Cat#RT1456;
Rabbit polyclonal anti-RAC1	Proteintech	Cat#24072-1-AP; RRID:AB_2879427
Rabbit polyclonal anti-SELENOW	Rockland	Cat#600-401-A29S; RRID_2285666
Rabbit polyclonal Laminin B1	Rabbit	Cat# A4373, RRID:AB_2863252
Mouse monoclonal MYH1 (Type I)	DSHB	Cat# BA-D5, RRID:AB_2235587
Mouse monoclonal MYH2 (Type IIA)	Mouse	Cat# SC-71, RRID:AB_2147165

Abberavations: 4EBP1, 4ukaryotic translation initiation factor 4E (eIF4E)-binding protein 1; AKT, protein kinase B; Atrogin-1, F-box only protein 32; CDC42, cell division control protein 42; EIF4G, eukaryotic translation initiation factor 4 G; FOXO3, forkhead box O3; GAPDH, glyceraldehyde 3-phosphate dehydrogenase; mTOR, mammalian target of rapamycin; MuRF-1, E3 ubiquitin-protein ligase TRIM63; MyHC, myosin heavy chain; P70S6K, ribosomal protein S6 kinase beta-1; RAC1, Ras-related C3 botulinum toxin substrate 1; SELENOW, selenoproteins W; Type I, myosin heavy chain Type I; Type IIA, myosin heavy chain 2A.

Table S3 Primer sequences of q-PCR reaction

Gene name	Forward primer (5' to 3' direction)	Reverse primer (5' to 3' direction)
Atrogin-1	CAGCTTCGTGAGCGACCTC	GGCAGTCGAGAAGTCCAGTC
MuRF-1	ACCTGCTGGTGGAAAACATC	CTTCGTGTTCCTTGCACATC
MyHC	ATAAAGGGGCTGGAGCACTG	AGGCAGGAAGAGGAGTAGCA
RAC-1	CCCAATACTCCTATCATCCTCG	CAGCAGGCATTTTCTCTTCC
SELENOW	AACATGAGTTCCCCGGATGC	CCAACTTCCCGGCTACTGTC
Rho protein signal transduction related gene		
ARHGEF40	CACAGGTCCAGAGTGGTTCC	GCTCGCCCAGTCTGATCTAC
EPS8L2	GGCTATCGCAGGAGGGAATC	TTCCCCTTTTTCCGCTGGTT
FGD5	GCCCTCCACTTAACCAGCAT	TCATTCTCTGGCACGACCAC
MCF2L	CAAGGTTTCCGGGAGGTCAA	TCCTCAAAGGCGGTCAAGTC
TIAM1	CAAAACAGGGACAGGACGGT	ATTCTCGGACGCTGAAGAGG
Muscle system process related genes		
MYOD	CTGCTCTGATGGCATGATGG	GTTCCCTGTTCTGTGTCGCT
MYOG	TGCCCAGTGAATGCAACTCC	TTGGGCATGGTTTCGTCTGG
CHRNG	TCCGACTCCAGAATGGCTCT	GCCTGCACTAATCCATTGCG
MYL4	TGCATCAACTATGAAGCCTTTGTC	TCCAAGCTGGGGCTCTTTAT
MYL6B	AGCAACGGCTGCATCAACTA	TTGCCTCTCAAGCGGATACC
MYOZ2	ACAGATCCCAGGTTCTTGGC	CAGATACCCACCCCTTTGGC
P2RX4	TGTGGCTGTGACCAACACTT	GCGTCTGAATCGCAAATGCT
TNNC1	GCTCATGAAGGACGGTGACA	CCGTGCAAGACCAGCATCTA
Control protein		
GAPDH	AGAACATCATCCCTGCATCC	GGTCCTCAGTGTAGCCCAAG

Abberavations: ARHGEF40, Rho guanine nucleotide exchange factor 40; CHRNG, cholinergic receptor nicotinic gamma subunit; GAPDH, glyceraldehyde-3-phosphate dehydrogenase; EPS8L2, EPS8 like 2; FGD5, FYVE, RhoGEF and PH domain containing 5; MCF2L, MCF.2 cell line derived transforming sequence like; TIAM1, MYOD, myogenic differentiation; MYOG, myogenin; MYL4, myosin light chain 4; MYL6B, myosin light chain 6B; MYOZ2, myozenin 2; P2RX4, purinergic receptor P2X 4; TNNC1, troponin C1; TIAM Rac1 associated GEF 1;

Table S4. Different expression genes in TA muscle between WT and KO aged mice

Note1: The gene expression level was measured by RPKM (reads per kilo base per million mapped reads). Values of mRNA levels of differential genes between the two groups are mean, n =3.

Note2: The raw data of RNA-seq reported here are deposited in the Gene Expression Omnibus database under accession no. GSE232366 (If can’t access, the private access token: ctofoekqzpwhxcz).
